# Illuminating the empowerment journey of caregivers of children with disabilities: Understanding lessons learnt from Ghana

**DOI:** 10.4102/ajod.v9i0.705

**Published:** 2020-11-27

**Authors:** Maria Zuurmond, Janet Seeley, Tom Shakespeare, Gifty G. Nyante, Sarah Bernays

**Affiliations:** 1 International Centre for Evidence in Disability, London School of Hygiene and Tropical Medicine, London, United Kingdom; 2 Department of Global Health and Development, London School of Hygiene and Tropical Medicine, London, United Kingdom; 3 Department of Physiotherapy, Faculty Biomedical and Health Sciences, University of Ghana, Accra, Ghana; 4 School of Public Health, Faculty Medicine and Health, University of Sydney, Sydney, Australia

**Keywords:** caregiver, carer, children with disabilities, empowerment, support groups

## Abstract

**Background:**

Empowerment is an increasingly popular goal, considered core to a transformative agenda for children with disabilities and their families. However, it can still be a poorly understood concept in practice.

**Objective:**

This article is an empirical analysis of the ‘empowerment journeys’ of caregivers participating in a community-based training programme in Ghana.

**Method:**

In-depth interviews were conducted with 18 caregivers at three time points over 14 months. Thematic analysis was conducted on the full data set, with three representative case studies selected for more detailed analysis to illustrate the dynamism of time and context in shaping the empowerment journey.

**Results:**

Our findings illuminate the complexity and non-linearity of the caregiver empowerment journey. There were important gains in individual dimensions of power and the nascent emergence of collective power, through improved knowledge and valuable peer support from group membership. However, further gains were impeded by their limited influence over wider economic and sociopolitical structural issues that perpetuated their experiences of poverty, stigma and the gendered nature of caregiving. The support group facilitator often played a valuable brokering role to help traverse individual agency and structural issues.

**Conclusion:**

A richer and more nuanced understanding of caregiver empowerment in the community and family context can inform the wider discourse on disability. Guidelines on working with people with disabilities, and the role of empowerment, should not neglect the pivotal role of caregivers. There are important lessons to be learnt if we want to improve family-centred interventions and transform the lives of children with disabilities.

## Background

Whilst definitions of empowerment are diverse, it is generally agreed that it is a process, or outcome, that is multidimensional and seeks to shift prevailing power dynamics, which can be at the level of people, communities or organisations (Luttrell et al. 2007).

Empowerment as both a process and outcome for families of children with disabilities is seen as increasingly relevant. The pivotal role that families play in improving health outcomes for women, children and adolescents is outlined in the Global Strategy on Women’s, Children’s and Adolescents’ Health (World Health Organization (WHO) 2016). This strategy calls for a transformative approach in which women and children can be the most ‘powerful agents for improving their own health’, through developing their own individual potential to make informed decisions, combined with active partnership with other stakeholders.

Empowerment is foundational to disability-inclusive development and community-based rehabilitation (CBR). It is one of the five pillars of the World Health Organization CBR matrix alongside the health, education, livelihood and social sectors. Empowerment is also a cross-cutting theme, and the guidance promotes the ‘importance of empowering people with disabilities, their family members and communities … to ensure that everybody is able to access their rights and entitlements’ (WHO, UNESCO, ILO & IDDC). Empowerment is also a core element of the International Classification of Functioning, Disability and Health (ICF), a biopsychosocial model of disability, where disability is conceptualised as the product of an interaction between bodily function and personal and environmental factors. Personal factors include elements of individual empowerment, such as self-esteem and resilience, and equally an environment that facilitates empowerment is essential (Shakespeare & Watson 2001; World Health Organization & World Bank 2011).

### Concepts and theories of empowerment and power

Much of the early conceptualisation of empowerment stems from the work of educationalist Paolo Freire; his work was essentially about the fight for social justice through social transformation, driven by power acquired through acquiring knowledge and resulting in the *conscientisation* of the individual, allowing them to drive change in their own lives (Freire 1996; Luttrell et al. 2007). These ideas were then heavily drawn upon in the discourse of how power could address poverty reduction in international development and establish that poorer people, through participatory empowering processes, are enabled to take more control over their lives (Chambers 1983, 1994).

Expanding on the work of Chambers, in the dialogue on gender and development, emphasis was given to the value of different dimensions of power, notably the personal and inner dimensions of power, as well as the need to examine the underlying structural drivers of oppression (Moser 1989; Rowlands 1997). Rowlands (1997) made a case for a more nuanced understanding of power, arguing that the earlier work on power (Foucault 1982) did not allow for factors that might influence an individual’s agency to act, or the idea of collective agency, and that previous models did not shed enough light on the social mechanism of power. Instead, based on a gender analysis of power relationships, Rowlands proposed a three-dimensional empowerment framework, exploring power at the personal level, within close relationships and at a collective level. Power is then divided into four categories: (1) *power within*, which is about individual capability and self-worth; (2) *power to*, which is about the agency of the individual to take actions; (3) *power over*, which is about an individual’s ability to access or influence economic, social or political factors; and (4) *power with*, which is about collective power to take actions with others (Luttrell et al. 2007; Rowlands 1997).

A parallel theory development was taking place in psychology in the 1980s on psychological empowerment (PE), focus on the individual and encompassing perceptions of personal control, a proactive approach to life and a critical understanding of the sociopolitical environment (Perkins & Zimmerman 1995; Zimmerman 1995; Zimmerman & Warschausky 1998). This model has three elements – intrapersonal, behavioural and organisational – with levels of empowerment varying across different life domains, for example, someone might be empowered in the home setting but not in the work setting, or vice versa.

At the same time, within the disability movement, the social model of disability placed an emphasis on removal of the structural barriers in society, in order to empower people with disabilities to overcome their experiences of oppression (Shakespeare 2006).

An additional important conceptualisation of power, found in Gaventa’s power cube (Gaventa 2005), offers a different lens for understanding the complexities of power. He describes power as being on a continuum, with categories of visible, hidden and invisible power. ‘Visible power’ is described as observable decision-making dictated by formal rules and structures, ‘hidden power’ describes which people and institutions get to the decision-making table, whilst ‘invisible power’ is described as more ‘insidious’ and is the power that shapes the sense of self, influenced by social and cultural norms that can perpetuate what is considered normal and acceptable. The model describes how these forms of power must also be understood in terms of the spaces and places (from local to global) in which power might be exercised, coming together in a three-dimensional power cube. He argues that it is insufficient to just be in possession of power, but people must have the space to then exercise power.

A persistent debate that runs through all these theoretical discussions on power and empowerment relates to individual agency versus a structuralist approach to change. The structuralist perspective proposes that empowerment approaches should be primarily aimed at dismantling social, economic and institutional barriers to have greater influence over change, and the human agency perspective places a greater emphasis on individuals’ capability to act rationally and autonomously (Baber 1991; Fazil et al. 2004). Instead, there is increasing recognition that both elements need to be present, are seen as complementary and dynamic forces (Luttrell et al. 2007) and are not binary; rather, there is a more fluid dynamic in how power operates. A review of the role of individual agency versus structural approaches in human immunodeficiency virus prevention concluded that this dichotomy was not helpful but that instead there needs to be a better understanding of the communities in which people act and connect in order to effect change (Kippax et al. 2013).

### Operationalising empowerment

Since this theoretical development, ‘empowerment’ has become a ubiquitous term and an increasingly popular buzzword (Cornwall 2007). Some argue that as a result it is now a devalued term that has been hijacked and depoliticised from its original meaning (Batliwala 2015). Despite its common use in health programmes, there continues to be limited clarification of the meaning and operation of ‘empowerment’ (Cornwall 2016; Crivello et al. 2014; Luttrell et al. 2007), and approaches focus too narrowly on individual change, such as adopting healthy lifestyles and improved self-efficacy of the individual (Laverack 2009).

Given the importance of empowerment in the CBR guidelines, it is also surprising that there is a dearth of literature on defining, understanding and measuring empowerment within programmes with people with disabilities in low-income settings (Rule 2013). Specific literature on caregiver empowerment is largely absent from the literature. In high-income settings, the lack of studies on what empowerment means in practice for family-focussed disability programmes has been highlighted, with a tendency for programmes to define and measure individual empowerment of the parent as an outcome of disability service provision (Banach et al. 2010; Nachshen 2005; Singh et al. 1995). In the United Kingdom, it has been argued that carer empowerment has received very little attention (Larkin & Milne 2014) and that too often the role of the mother is undervalued and peripheral in the discourse about children with disabilities (Ryan & Runswick‐Cole 2008). A study of an empowerment and advocacy programme in the United Kingdom with caregivers argued that insufficient attention is given to the social, cultural and familiar contexts and other structural issues that can limit the capacity to change (Fazil et al. 2004). Despite the popularity of the term, with some exceptions (Joseph 2020), it continues to not receive much critical attention.

Given this critique around the operationalisation of empowerment, and limited research on caregiver empowerment in the lives of children with disabilities in low- and middle-income settings, where arguably there is more dependency on families to provide most of the care, this article sought to examine the experience of empowerment of caregivers who engaged in a 1-year training programme in Ghana.

## Methodology

### Intervention

This article draws on data from a large pre- and post-intervention study to evaluate the impact of a caregiver training programme called Getting to Know Cerebral Palsy (LSHTM & Hambisela 2013). This was a 1-year programme, with 10 modules that were participatory in nature to promote critical thinking, problem-solving and peer support, based on principles of adult learning theory (Knowles 1984). The parent support groups were established by the local implementing partner, the Presbyterian Church of Ghana, in sites where they worked, and had an infrastructure for CBR or inclusive primary healthcare programmes. Up to 10 parents per area were invited to join a support group and participated in 3–4-hour training sessions on a monthly basis, with topics that included *understanding your child, communication, evaluating your child, play, eating, disability in your community, running your own parent group* and *everyday activities*. Referrals were also supported for assistive devices. Each caregiver had a child aged 18 months to 12 years with a confirmed diagnosis of cerebral palsy.

Caregivers also received a monthly home visit from a group facilitator and a community session to raise awareness about the programme. The groups were run by a pair of facilitators who were therapists, normally a local physiotherapist assistant combined with a primary healthcare worker such as a special needs teacher, nutritionist or a CBR worker.

The impact of the programme on well-being has been published (Zuurmond et al. 2018a, 2018b). In the broader study, 75 primary caregivers were invited to join a caregiver–parent support group in one of eight districts in Ghana. The primary caregiver was defined as the member of the family with the main responsibility for looking after the child. In this article, the research questions we seek to explore are: (1) to understand the role of empowerment of caregivers as they engaged with the training support programme and (2) to understand the key factors that shaped caregiver empowerment, at the level of the individual, family and community ecosystem.

### Participant selection

**FIGURE 1:**
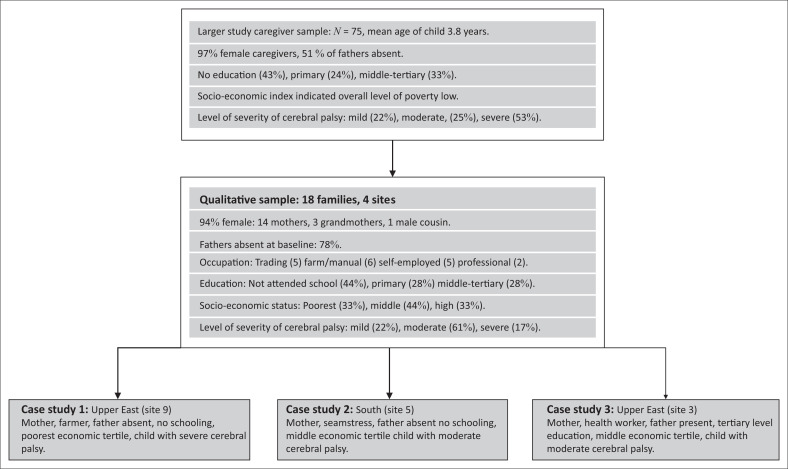
Details of sampling process.

Caregivers were identified through the community-based screening programme for cerebral palsy and through the hospital records of children diagnosed with cerebral palsy in the last 6 months. For the in-depth qualitative study, 18 families were then purposively selected from four sites, to ensure a geographical spread, different socio-economic status and a mix of children according to gender, age and severity of cerebral palsy. Eleven families were initially selected, and following the death of three children, a further five families were selected in the second round of interviews and two more at end line. Participant selection details are illustrated in Figure 1.

### Data collection

A total of 37 in-depth interviews were conducted with 18 primary caregivers across three time points: 2 months before the start of the training programme; around 6 months into the training; and within 1 month of completion of the programme. Semi-structured interview guides were used, and all interviews were conducted in the home. The guides initially explored issues of what the child was able to do, what their understanding was of the condition, and who provided support within the family and questions about the caregiver well-being. Mid-term questions probed engagement with the programme and changes experienced. Topics also emerged through a process of iterative data collection and analysis in which areas of further investigation were developed in light of emerging ideas and concerns expressed in the interviews. Supplementary shorter interviews were conducted with selected secondary caregivers at the time of the household interviews, in order to capture additional perspectives on the caregiving experience within the household, and detailed field notes were kept. The interviews were conducted either by a local Ghanaian or by an international researcher (female Ghanaian, G.N.; white British female, M.Z.). Interviews were conducted in four local languages with translation into English as required. All interviews were audio recorded, translated into English and then transcribed.

### Analysis

Two key stages of the analysis were conducted: a thematic analysis across all data from the 18 families at baseline, mid-term and end line and then a biographical case study analysis, which collated all the data from each family into a case study and detailed the change over time for each of the 18 families. The data included transcripts, as well as field notes and project monitoring forms, in order to provide a more holistic overview of their lives, in line with the guidance for longitudinal analysis (Creswell 2013; Green & Thorogood 2009).

For purposes of better illustrating the change over time, we are presenting three case studies. The case studies were selected to be representative of the larger sample (see Figure 1) and to illustrate and explore pertinent thematic concerns. Focussing on fewer individuals enabled us to obtain greater richness, detail and completeness than with other analytical approaches (Flyvbjerg 2013, Prior 2016) and helped us better illuminate the influence of the dynamic relational, social and economic context over time, which is not always captured so clearly by presenting a thematic analysis.

We used the conceptual framework of Rowland’s model of power (Luttrell et al. 2007), as detailed in Table 1, to explore power across four different domains. We also applied the socio-ecological model (Bronfenbrenner 1994), which outlines the multiple ecosystems in which children and their caregivers are embedded, thereby exploring the domains of power at the individual, family and community levels.

### Ethical consideration

Ethics approval was obtained from the Noguchi Memorial Institute for Medical Research, University of Ghana, and from the London School of Hygiene and Tropical Medicine (reference number: 8905; 25 March 2015), United Kingdom. Informed written consent was obtained from all participating caregivers, with a signature or thumbprint. All children identified with malnutrition were referred for follow-up, and the CBM child protection policy was adhered to. The case studies have all been provided with pseudonyms in this article.

## Findings

### Our case study families

Seventeen of the 18 participants were women: 14 mothers, 3 grandmothers and 1 male cousin. The overall level of caregiver education was low, with eight never having attended school and only three having attended high school or tertiary education. A socio-economic index illustrated that most families were extremely poor, and fathers were completely absent, lived separately or worked away from home, commonly with infrequent visits.

In summary, from the thematic analysis, the key emerging themes across the data from all 18 families were (1) acquisition of *power within* at the intrapersonal level, (2) the gradual development of *power with* other group members, (3) the brokering role of the group facilitator and (4) the economic and sociopolitical structural issues that very often limited the caregiver’s *power over* change. The intersectionality of power with gender, poverty and stigma was also evident.

We organise our results by firstly presenting a case study and then linking the case study to the wider thematic analysis conducted on data from across all 18 families.

#### Case study 1: Jacinta and Maxwell

Jacinta, a single unmarried mother with two children, lived with her own mother in rural Upper East Ghana.

One of her sons, Maxwell, was 2.5 years old, had severe cerebral palsy and was severely underweight and stunted when we first met him. The grandmother had elephantiasis and had limited mobility. They were subsistence farmers, with some small additional income from hat weaving. The mother was unmarried, and the father of the children visited once over the 14-month period, bringing soap as a contribution to the household.

When we first met Jacinta, before she joined the support group, she did not raise her eyes from the ground. It was her mother who provided detail about how difficult their situation was, the particularly high levels of stigma experienced because of traditional views about the child and how isolated they felt. When we met Jacinta 6 months after attending the group, she laughed and chatted openly about the programme and talked of new skills acquired to improve the care for her son. She had felt confident enough to explain her son’s condition to neighbours:

‘Before, they [*neighbours*] used to insult me that I have given birth to a *Kinkiriku* [*spiritual child*]. They used to say this to the child: “Go away you, this Kinkiriku.” That was before I knew the group. After I met the group, I always could explain to them what I learnt. Now they do that no more.’ (Jacinta, code 9916)

Jacinta reflected on feeling valued as a ‘human’, having status conferred by the value of her group membership and meeting other mothers who shared the same situation, thus building her social capital, and also through feeling valued and worthy enough to be visited at home by a facilitator. Jacinta’s case illustrated the development of the *power within* as she gained more self-confidence, self-esteem and feelings of self-worth:

‘At first my mum and I used to weep. I thought I was the only one with this problem but when I saw my colleague women with similar problems, I realised that I wasn’t the only one with this problem.

I feel that we are also human beings and that is why people have come to visit us. Their coming makes me happy.’ (Jacinta, code 9916)

In terms of *power to*, Jacinta was able to comment on her various improved caregiving skills, and she felt able to share that information with her biological mother, thus reducing her own caring workload. Despite these positive changes at the individual level, a lack of political and economic power remained a major impediment to Jacinta when we met her after 6 months. She had run out of cash for hat-weaving, and poverty was a major challenge, exacerbated by her inability to work away from home because of the need to look after her son. The family did not benefit from any social protection initiatives, such as the LEAP (Livelihood Empowerment Against Poverty) programme, because as they explained, they lacked political allegiance to community leaders: ‘If you are not in their politics … you wouldn’t be picked’. This demonstrated the invisible and hidden aspects of power that exist in communities and the lack of space made available to our caregivers to exercise their power. The group facilitator has good contacts with local community health workers, and he now facilitates Maxwell’s inclusion in a nutritional programme, whilst Jacinta had previously been turned away.

This case study illustrates a prominent change across all caregivers, that is, improvement in their *power within*, over the 1 year. The solidarity of the support group was a common theme, frequently described as ‘like a family’, and the realisation that they were ‘not alone’ appeared to play an important role in their empowerment journey. Another common theme illustrated, and shared across most interviews, was a reduction in self-blame, generated from having more knowledge about their child’s condition, thus helping with improved feelings of self-worth. Whilst Jacinta’s case study illustrated the *power to* change her caregiving practices and share knowledge and skills with her own mother, the broader thematic analysis reflected mixed caregiver experiences. Frequently, relationships at the family level remained strained over the year, especially within the husband’s family, and mothers continued to have little power within the social norms and power structures.

#### Case study 2: Beatrice and David

Beatrice was a confident and articulate young mother when we first met her. She had two children and lived on her own in the outskirts of Accra, renting one small room. Her son, David, was 4 years old and was diagnosed with severe cerebral palsy. Beatrice was a seamstress by trade, but because of full-time caregiving for her son, she was not working when we first met her, and this was a source of financial problems. Her husband left them shortly after her son’s birth, blaming Beatrice for ‘bringing disability into the family’, and the last time he had visited was more than 3 years ago. He provided no support.

When we met Beatrice a second time at 6 months, there was growth of her *power within* and increasing evidence of *power to* take actions. She was positive about her newly gained knowledge, had implemented improvements in caring for her son and had taken steps to enrol David in school. However, she was upset about how she had been treated by the head teacher, who turned her away and said the school was unsuitable for her son. The group facilitator was looking for another school. In terms of catalysts and impediments to empowerment, it was evident that the group facilitator played a vital role, using his own position as a special needs teacher and his networks to negotiate the ‘hidden’ and ‘invisible’ power of the education system, and being offered a space at the table to exercise that power. He finally secured a place for David at another school.

When we met Beatrice for the third time, after 14 months, the *power with* other group members to take collective action was slowly materialising. For example, several group members visited a mother who needed extra support in facing a difficult situation at home. In the absence of other community support mechanisms, the group ‘family’ appeared to be playing an increasingly valuable role as a social safety net for many of the caregivers. Sadly, Beatrice explained that she had ‘regressed’ since we last saw her, mainly because she had obtained a job as a seamstress, but then had lost the job and borrowed money to try a variety of small trades, all of which had been unsuccessful and resulted in debt. The most significant impediments to Beatrice’s empowerment journey were her lack of economic power and her struggle to meet even the basic needs necessary for survival:

‘There are days that I struggle to get something to eat and I sleep on an empty stomach. It is not just once. And I don’t want to be a burden on the people I live in the house with … the little I have I give to the children.’ (Beatrice, code 5558)

This case study illustrates the very common impact of poverty on caregivers’ agency across the sample, with the exception of the only two mothers who were in regular paid employment. Poverty was exacerbated when a mother lived unsupported by the child’s father or his family, as was the case for almost all parents in our sample. Even where mothers were living in extended families, a common theme was exclusion and a lack of power over economic resources within the household, which limited their power to take simple actions, such as buying more nutritious food for their child or taking their child for necessary health checks. As the local CBR manager reflected, for many of the families, ‘empowerment starts with the stomach’.

The ‘brokering’ role of the facilitator in helping caregivers navigate their way was a recurring theme across all families, such as the facilitator helping to renew a health insurance card, to negotiate the administration of access to the Disability Common Fund, to help organise equipment repairs or to facilitate access to health or education services. Although the caregivers had acquired knowledge and confidence, social and political processes were still sometimes overly complex to navigate, or caregivers were not afforded a space to exercise their power. This was illustrated by one mother who finally had the confidence to go to the government office to register her daughter for the Disability Common Fund, only to be turned away.

#### Case study 3: Carol and James

Carol was educated to the secondary level and was one of only two mothers in the sample to have a professional job with a regular income; she was an administrator in the government health service. She was the only mother who had a husband living at home and who did not work away, and she had two daughters and a son. Her son, James, was a very bubbly smiling boy of 4 years old, with moderate cerebral palsy. Her son was turned away from the local government school because of his disability, but she chose a private school for him and drove him to school on her motorbike.

The first time we met her, before she started the programme, she described her fight to get a diagnosis for her son. After visiting various doctors over a 2-year period, she finally used her work network to approach the Regional Health Director to demand a diagnosis. This demonstrated high levels of self-confidence from the outset, actively seeking information and support. As the main breadwinner in the family, she had power over economic resources, and this facilitated her decision to send her son to private school and to obtain extra healthcare for him. Her case study mirrored the one other mother who was also in paid employment.

When we met Carol at 6 months, this *power within* had translated into her becoming a key mother within the support group, supporting the facilitator with running the group, and someone that other mothers turned to for support and advice. Although *power with* activities were still limited outside of the group, there were valuable examples of organising collective visits to each other’s homes across all the groups:

‘We are more like a family now; we share, we do everything together. When one is having problems, we look how to solve it, and when there is always a problem. I am always helping Fred [*the group facilitator*], and so we are always looking for a way to solve it.’ (Carol, code 3340)

However, at 12 months, she tearfully explained that her husband was imprisoned, and she was struggling to hold down her full-time job whilst caring for all three children. She was also 6 months pregnant. Whilst she was happy that her son was making progress, and importantly was almost able to stand, she had recently come to realise that James had substantial visual impairment. This was a shock to her, that she was coming to terms with, and she was tearful and concerned that he might need to enrol in a school for the blind. She was looking for support and guidance from the group facilitator to help her navigate the educational and treatment choices for her son.

This case study illustrates the complexity of the empowerment journey, and mirrors the complexity of all the caregiver lives in this study, with the fluidity of changing support needs over time. In Carol’s case, it was her husband’s imprisonment, and the changing care needs of her son, that impacted on her empowerment journey. For other mothers, it was shocks such as being forced to leave rented accommodation because of the stigma associated with their child’s condition, a husband moving out to look for a second wife or the loss of work because of their caregiving duties.

## Discussion

The article explores the journey of caregiver empowerment and the factors that shape this process for caregivers of children with disabilities. This offers a critique of the narrow understanding of what it means to ‘empower a family’ when the focus remains limited to the individual. Our analysis indicates the pertinent influence of broader relational and structural conditions in impeding the impact of an empowerment programme on the lives of caregivers and their children.

Instead, we offer a more nuanced understanding of that journey as caregivers engage with a community-based participatory training programme in Ghana. We found the metaphor of a journey useful, as proposed by Cornwall (2016), to describe the empowerment process. The pathway is wide; some terrain is easier with gains in some dimensions of power, whilst other paths are more difficult to traverse, and they may need more help. There are also a variety of routes, reflecting the non-linear nature of their journey, as caregivers respond to changes in their often-precarious lives, including the changing care and support needs for their child. Reynold’s model of empowerment provides a useful framework for illuminating the different dimensions of caregiver empowerment.

In our study, it was evident that the individual *power within* was strengthened through the support group training. The value of this dimension of power should not be underestimated, given the profound levels of stigma and discrimination that are commonly experienced by caregivers, primarily mothers, for having a child with a disability (World Health Organization & World Bank 2011), and specifically the high levels of self-stigma and self-blame that are common to this group (Nyante et al. 2017). It may be that without developing power within, it will be difficult to achieve other aspects of power, but we also argue that the reduction of empowerment to individual agency alone is too limited.

The findings also illustrate, within the family ecosystem, that the *power to* take decisions and actions was facilitated or impeded by the hierarchy of kinship structures and the gendered nature of caregiving. This aligns with the need for greater understanding of the nature of human relationships in society and how that influences power (Green 2018). Additionally, within empowerment theory, there is a need to strengthen our understanding of how disability-related stigma plays out within the family context and interacts with power dynamics, within any model of empowerment.

In terms of *power over* economic and political resources, the linkages between poverty and disability are increasingly well documented (Banks & Polack 2014; Groce et al. 2011), but this literature is often focused on the adult with a disability, with less evidence of the impact of poverty on children and their caregivers. In Maslow’s hierarchy of basic needs, he argues that physical survival needs must be met first before people can feel belonging and self-esteem and maximise their self-potential (McLeod 2007), and therefore operationalising empowerment for caregivers requires better understanding of the intersectionality with poverty.

We would argue that important changes occurred in caregiver feelings of self-worth and self-esteem that were essential stepping stones on their journey, but extreme poverty still limited their self-potential, when the main concern for some was putting food on the table. In the context of poverty, life is precarious for any family, but this research illustrates the augmented vulnerabilities brought about by having a child with a disability and how this shapes the possibilities for empowerment.

Therefore, drawing together these different experiences, our study shows that if we want to take a more transformative approach to maternal and child health for children with disabilities, it requires approaches that permeate the outer circles of the child and caregiver ecosystem. This is about a closer alignment with empowerment theory, which was always about looking at different dimensions and levels of empowerment and never only about the individual, even if that is more difficult for us to evaluate. There can be a tendency in the framing of family-focussed disability programmes, ours included, to lean more on the psychological framing of empowerment, about fostering change in the individual caregiver, but this study shows that it is about relationships, networks and structural issues.

Although not a focus of the initial programme, on completion of the study, the caregivers engaged collectively on a local advocacy activity through the local community radio to raise awareness about their issues. Other studies have similarly shown the power of collective agency for change by caregivers of children with disabilities (Elphick et al. 2015) and for improving community-level maternal health traditional practices (Badas et al. 2011; Morrison et al. 2010). This is an important element that could be strengthened within the future development of our programme. A study of self-help groups for caregivers of children with disabilities in Kenya also demonstrated that caregiver empowerment was associated not only with newly developed skills but also social connectedness and resource mobilisation (Bunning et al. 2020).

If we return to theories of empowerment, we would argue that it is also valuable to look at who can play a key role in the reorientation of power within the ecosystem layers. In our study, the group facilitator played a crucial role in brokering power over local social and political processes and thereby facilitating easier steps for caregiver empowerment. Local community health or education professionals are part of the invisible and hidden power structures (Gaventa 2005) and thus are often better positioned to engage with these processes. There should not always be the expectation that caregivers, mainly women, can always be the prime agents of change, given that a range of factors coalesce around caregivers, including poverty, stigma, poor levels of education and gender, which may limit their ability, even collectively, to engage with social and political power processes.

At the same time, we also recognise the possible tension with creating dependency on a facilitator, and this is similarly explored in a critique of disability care in Africa (Morvan et al. 2014), or with CBR workers, who do not necessarily have the skills to foster empowerment (Rule 2013). This aligns with the argument made to enhance facilitator skills with a ‘fifth power’, the ‘power to empower’, as proposed by Robert Chambers (2012).

Finally, our study also calls for an improved understanding that the empowerment process is not always a linear one, especially when lives are so fragile. And given this fragility, and the long-term support needs for children with developmental disabilities, the empowerment journey is never likely to be a quick process; caregivers will have different support needs at different times. This is summed up in a discussion on women’s empowerment: ‘when we find the path we wish to tread, first walk in front of us; then, when we are stronger, walk beside us; and finally, when we are truly strong, walk behind us’ (Batliwala 2015). We therefore need to be more realistic about operationalising theories of empowerment in practice for parenting programmes in low-resource settings.

## Limitations of study

Future research would benefit from observations of the support group and of the home visit and understanding more about the role of the group facilitator. It would also benefit from revisiting the families after a longer time period in order to understand how some changes have evolved and are sustained. The families in the study were in areas supported by the local partner, the Presbyterian Church of Ghana, which typically works in areas of greater deprivation, and many of our families were the ultra-poor, where poverty was likely to play a greater role and where there were very low levels of caregiver education. Ultimately, this training is intended to improve outcomes for children with disabilities, and as such, children should also be taken on an empowerment journey. In this study, the majority of the children were under 5 years, but in any future study with older children the issues of children’s empowerment should be explored.

## Conclusion

New global maternal and child health strategies call for a more transformative approach, with women and children as agents of change. Empowerment is also core to the WHO CBR guidelines. Despite much work over the years on empowerment as a theoretical construct, research on how this works in practice for families of children with disabilities in low-resource settings is limited. We illustrate some important gains in caregiver empowerment as caregivers engaged with a participatory training programme in Ghana, in particular in terms of improvements in *power within* the individual. However, there are also multiple ways in which caregivers do not have access to decision-making; the gendered nature of caregiving, the intersection with poverty and disability-related stigma are key factors that limit their agency. We illustrate the lack of power over sociopolitical processes and the potential benefit of someone who can play a brokering role. There are limitations to any approach that places too much emphasis on individual agency and improved self-efficacy. A strengthened intervention needs to permeate the layers of the ecosystem in which the caregiver–child dyad is embedded, combined with addressing structural issues to foster a more enabling environment. There are important lessons to be learnt if we want to transform the lives of children with disabilities, and their families.
